# Medical schools in empires: connecting the dots

**DOI:** 10.1017/mdh.2024.14

**Published:** 2024-04

**Authors:** Hohee Cho, Martin Robert

**Affiliations:** 1 Faculty of History, University of Oxford, 45-47 Banbury Road, Oxford, OX2 6PE; 2 Centre for History in Public Health, London School of Hygiene and Tropical Medicine, 15-17 Tavistock Place, London, WC1H 9SH.

**Keywords:** Medical education, Medical school, Colonial medicine, Empire, Doctor, Medical profession

## Abstract

This article provides an overview of the historiography of medical education and calls for greater attention to the connections between medical schools. It begins by reviewing research on medical education in imperial metropoles. Researchers have compared medical schools in different national contexts, traced travellers between them or examined the hierarchies that medical education created within the medical profession. The article then shows how historians have emphasised the ways in which medicine in colonial empires was shaped by negotiation, exchange, hybridisation and competition. The final part of the article introduces the special issue ‘Medical Education in Empires’. Drawing on a variety of sources in English, French, Dutch and Chinese, the special issue builds on these historiographies by juxtaposing cases of medical schools in imperial contexts since the eighteenth century. It considers who funded these medical schools and why, what models of medicine underpinned their creation, what social changes they contributed to, what life was like in these schools, who the students and teachers were and what graduates did with their medical careers. This special issue thus contributes to clarifying the role of medical education in empires and the long-term impact of empires on the medical world.

For seven Korean medical students, June 1908 marked the end of their studies.^1^ In their hands were Korea’s very first state-recognised medical licences, awarded during a ceremony at Severance Hospital Medical College in Seoul. Wearing Western gowns over their traditional Korean attire, the graduates—who all came from different class backgrounds—were presented with ceremonial caps by the school’s British-Canadian headmaster, Oliver R. Avison. Each graduate received his title from Itō Hirobumi, the Japanese Resident-General, who was the Japanese government representative in charge of political affairs in Korea. This foreshadowed what lay ahead for the country. Two years later, Japan, which already controlled Korea’s diplomatic relations, would completely colonise it. Founded in 1885 and known as Je Joong Won until 1904, the College was the result of a collaboration between a Canadian doctor, Presbyterian missionaries from the United States and one of Korea’s last monarchs. It was the first educational institution in Korea to teach medicine inspired by European models.

The significance of the ceremony was underlined by the presence of nearly 900 guests, including senior politicians and Western envoys to East Asia. In addition to the Korean and Japanese flags that were waving were British, American, and Red Cross flags, signalling the origins of the institution’s sponsors and managers, and presumably in the spirit of affirming the universal humanitarian value of medicine.^2^ The ceremony was at once a display of Japanese imperial authority in a semi-colonial state, American missionary endeavour and British oversight represented by the faraway dominion of Canada. It was a global and imperial event, encapsulating the relationships between people from different colonial, imperial, missionary, humanitarian, medical and class backgrounds under a single banner: state-sanctioned medical education. Today, Severance Hospital is still a teaching hospital and is now affiliated with Yonsei University.

The hybrid nature of this event was in many ways typical of the two centuries following the Seven Years’ War (1756–63). At a time when colonial empires were poking their noses into every corner of the globe, teaching and healing were being massified and formalised behind the walls of various new schools, faculties, hospitals and institutes. The interpersonal relationships of the artisanal world that had characterised much of medical education until the eighteenth century were giving way to more impersonal interactions in the medical institutions that were springing up. More than ever before, medicine fed the desire for a universal science: one science for the whole world. This universalist ideal collided with the multiple ways of understanding and treating the human body, resulting in the interplay and fragmentation of ways of thinking, treating and behaving, sometimes in discord that could become violent. This was not so much the dawn of a global medical world. As medical schools popped up on every continent, what was taking shape in medicine was the emergence of specific, connected points around the world, or ‘webs of empire’ as Tony Ballantyne described colonial networks of knowledge.^3^ More specifically, the links between medical schools in Edinburgh, London, Calcutta, Montreal, Sydney and Cape Town, for instance, outlined the ongoing conflicts and worldwide ambitions of medical education in the British Empire. Those of the French Empire involved medical schools in Paris, Cairo, Bordeaux, Pondicherry, Hanoi, Shanghai and Dakar. Medical schools funded by the Rockefeller Foundation, for example in Baltimore, Beijing and Bangkok, reflected the major influence of this philanthropic institution on the medical world, much as the international network of Pasteur Institutes influenced the development of Pasteurian bacteriology. New models of doctors were emerging, more uniform but also more attuned to both an increasingly intercontinental labour and health market and their immediate environment.^4^

The aim of this special issue is to explore the history of medical education by juxtaposing cases of medical schools shaped by imperial ambitions since the eighteenth century. Why medical schools? Because they have become the dominant form of medical education in the world. They reveal what doctors are expected to be at a particular time and place. From a practical point of view, the analysis of the same type of institution allows for more meaningful comparisons between cases. Although our definition of a medical school is flexible, the institutions analysed in this issue share some characteristics. They offered a similar core curriculum leading to a legally recognised qualification. In contrast to private medical apprenticeships that remained common well into the nineteenth century, including in European imperial metropoles, the recruitment of students in medical schools was not primarily based on family connections or personal networks. Medical graduates also tended to occupy a subordinate position if they came from the local population in the colonies. Many medical schools established under imperial regimes outlived the individual careers of their founders, and some are still active.

And why empires? Because in empires the interplay between health and disease, the body and the environment, politics and economics, and culture and demography emerge in its greatest complexity, as in the British, French, Dutch and American cases examined here, which operated over vastly different territories and populations. Medical education in empires is a window on the elaboration and transmission of scientific knowledge and practices, global geopolitical conflicts, and the construction of trust and authority on the human body through institutional qualification.^5^ Looking at medicine through the window of medical education in empires is what we invite you to do here.

The growing historiography allows us to look more closely at medicine in imperial metropoles and in colonial territories. Let us look first at the historiography of medical education in the imperial metropoles and then at the growing body of work on medicine in colonial situations to see how they can help us understand medical education in empires.

## Medical Education in Imperial Metropoles

Much, if not most, of the historical scholarship on medical education has consisted of works about a single nation or institution at a time (faculty, school, institute, hospital, etc.).^6^ This is sometimes done in a commemorative perspective, with varying degrees of reference to the broader historical context. English and French publications on medical education have tended to focus on countries in Europe and North America. The United Kingdom and the United States are particularly well represented in this historiography, with a variety of studies of individual institutions, in addition to national overview studies, including the important contributions of Kenneth Ludmerer on the United States, Roy Porter and Vivian Nutton on the United Kingdom and the impressive four-volume history of the Royal College of Physicians of London by George Clark, A. M. Cooke and Asa Briggs.^7^ The latter explores what it meant to be a member of the medical profession in the United Kingdom between 1518 and 1983, analysing the interaction between the professions of general practitioner, specialist doctor and allied health professionals such as surgeons, obstetricians, gynaecologists, nurses, pathologists, psychiatrists and radiologists.

On the basis of this historiography, the general pattern over time appears to be that medical training became somewhat more uniform between the eighteenth and twentieth centuries, with an emphasis on bedside teaching, human dissection and, later, laboratory medicine. This varied according to the institution, regional differences and changing circumstances.^8^ At the same time, much of the research of recent decades in the history of medicine has questioned George Basalla’s model of a rather homogeneous Western science spreading from Europe and the United States to the rest of the world.^9^

To begin with, it is clear that ‘Western medicine’ has not been uniform. In Western Europe alone (or even in the United Kingdom), state-approved medical training was far from uniform until very late, if it ever was. Challenging this idea of uniformity in medicine, a whole branch of historiography has adopted a comparative approach to show the extent to which the systems of medical education in different European and American countries have either interacted with or diverged from each other. William F. Bynum’s *Science and the Practice of Medicine* examined how Britain, France, Germany, Austria, and the United States trained medical professionals in the late eighteenth and in the nineteenth centuries. Although each national context was different, these countries taught medicine in schools as a form of science, with the aim of instilling scientific thinking and a critical mindset in students.^10^ In a book chapter on the same topic, Bynum highlights the national characteristics of medical education in the nineteenth century. German-speaking countries, he argues, had a strong university tradition in the pursuit of systematic knowledge. Post-revolutionary France placed less emphasis on theory and more on hospital experience and post-mortem examination. The British model at institutions such as the University of Edinburgh or London hospitals like St. Bartholomew’s, Guy’s or St. Thomas’ emphasised bedside teaching. In the United States, proprietary schools were widespread.^11^ By comparison, Othmar Keel, in a book edited by W. F. Bynum and Roy Porter that includes six chapters on medical education, argues that several countries in Western Europe had some form of medical education in hospitals in the eighteenth century.^12^ In a similar vein, Thomas Neville Bonner compared and contrasted medical education in Britain, France, Germany and the United States from the mid-eighteenth to the mid-twentieth centuries. In chronological order, Bonner sketches a panoramic view of what was happening in medical education in Europe and the United States, providing one of the most coherent histories of this topic in the North Atlantic world to date.^13^

That said, the collective volume edited by Charles Donald O’Malley in California in 1970 under the title *The History of Medical Education* is undoubtedly one of the most ambitious attempts to write a general history of medical education throughout the world from antiquity to the twentieth century. This volume is the result of a symposium held in 1968. It offers a wealth of very useful studies of medical education in Europe, certain parts of Asia and the Americas. Few attempts have been made before or since to synthesise the history of medical education on such a scale.^14^ But as far as our approach is concerned, the studies in O’Malley’s volume are mainly based on nation-states, not empires.

Rather than taking a comparative approach, another strand of historiography has emphasised mobility and connections across borders, focusing on the travels of medical students and doctors. This has revealed preferred destinations at certain points in time and what might be called medical diasporas, either in the sense of graduates from the same medical school or faculty scattered around the world or in the sense of international migrations of doctors working in a country other than the one in which they were born and trained.

European medical students and doctors have long travelled to train and meet each other. This seems to have been particularly important since the sixteenth century, as explored in the collection edited by Ole Peter Grell, Andrew Cunningham and Job Arrizabagala on medical professionals travelling between different parts of early-modern Europe.^15^ By the beginning of the nineteenth century, travelling for medical training had become much more widespread. The Channel between France and the United Kingdom, and the North Atlantic became major crossroads in this respect. Large numbers of students from Canada and the United States went to study in Paris or Edinburgh, for example. These two capitals became points of convergence for medical education, in large part because of the opportunities they provided for learning at the bedside in hospitals, at the dissecting table and in the operating theatre—the globally influential surgical innovations of Joseph Lister (1827–1912) at the end of the nineteenth century being a case in point.^16^ Incidentally, Lister’s contemporary, Louis Pasteur (1822–95), was no lightweight himself when it came to global influence, not least through the international network of Pasteur Institutes that still exists to this day and between which countless health care professionals have travelled to study and work. Different aspects of all this have been carefully analysed by historians, including Erwin H. Ackerknecht, George Weisz, Toby Gelfand, Lisa Rosner, John Harley Warner, Florent Palluault, M. Anne Crowther, Marguerite W. Dupree, Aro Velmet and Matheus Duarte Alves da Silva.^17^

In the late nineteenth century, Germany (and German-speaking universities) also attracted a large number of students from overseas, leading to German becoming one of the main languages of biomedical science.^18^ According to Godelieve van Heteren, the number of British ‘medical travellers’ visiting German-speaking universities jumped from 76 in 1851–60 to 391 between 1881 and 1890, whereas ‘tens of thousands’ of Americans were spread across Europe.^19^ Hoi-eun Kim has extensively studied the travels of medical students and doctors between the German Empire and Japan from 1869 to the First World War, showing how medicine under the Meiji regime and German medicine interacted closely at a time when both the German and Japanese states were engaged in a process of empire building that was redefining their medical education and health care systems.^20^ Concerning the twentieth century, research has also brought to light the travels of specific groups, such as American Jews in Scotland in the 1930s, or Poles in Edinburgh during and in the immediate aftermath of the Second World War.^21^

Overall, these journeys may have reduced national differences in medical education to varying degrees or, on the contrary, exacerbated them, but the fact remains that the medical world became more closely knit as a result of travelling. It has become even more so since then, given the development of increasingly powerful means of communication and travel.

In addition to comparative methods and those focusing on individual or collective travels, medical education in imperial metropoles has been analysed through the prism of the hierarchies it created. In particular, the doors to medical studies have long been closed to certain categories of people because of criteria that could be based on race or gender. This is typically the case for minorities in the United States, as analysed in this issue by Christopher D. E. Willoughby, or for women, as studied by Laura Kelly, Susan Wells and Tracey L. Adams in the cases of Ireland, the United States and Ontario, respectively.^22^ Douglas M. Haynes has recently explored how the centralised national registration of doctors served to control who could practise where within the British Empire from 1858 to the end of the twentieth century. This created an ambiguous dynamic in the metropole, sometimes of rejection, sometimes of attraction, towards doctors from colonial or post-colonial territories who wished to practise in the United Kingdom.^23^ Comparable dynamics have been analysed by Sasha Mullaly and David Wright in Canada.^24^ Such hierarchies were part of medical education itself. Medical pedagogy supported by imperial metropoles almost always involved categorising and racialising bodies and sometimes literally turning them into teaching specimens to establish scientific authority. Race and gender in medical education are now being researched in greater depth than ever, as, for example, demonstrated by Rebecca Martin’s work on the normalisation of skin whiteness in nineteenth-century British anatomical models.^25^

But instead of being entirely closed, the doors of medical education could be open with such restrictive parameters that sub-classes of practitioners were forced into strict professional dependency after graduation. Indeed, the counterpoint to the movement of doctors and medical students around the world was that certain practitioners, because of the place of origin or ethnicity with which they were associated, were tied to specific territories through the medical training they received. This was particularly the case in imperial contexts, in which classes of subordinate practitioners, such as vaccinators, were explicitly trained to serve particular purposes in colonial settings. Their qualifications were only recognised in a narrowly defined area, which prevented them from travelling to practise their profession elsewhere. Much like a passport, a medical degree or licence made it easier or harder to work abroad, depending on the medical school or regulatory body that issued it. Recent studies of the migration of doctors whose education provided the ‘passport’ to move across colonial or post-colonial borders, particularly between territories that were once part of the same empires, echo this reality.^26^ For example, although graduates of medical schools in Fiji, Uganda and Nigeria could only practise medicine in the region where they had been trained, the Colombo Medical College in present-day Sri Lanka and the Faculty of Medicine at the University of Hong Kong awarded degrees that could be registered in Britain. The Royal University of Malta and the King Edward VII College of Medicine in Singapore awarded degrees recognised by the General Medical Council in the United Kingdom but on the Colonial List.^27^ Australia, New Zealand and Canada also had reciprocal arrangements with Britain.^28^ This aspect of medical education in empires runs through our special issue.

To reflect this complexity, the history of medical education in empires requires longer timelines than those beginning with the *Flexner Report* of 1910, which is often seen as a turning point in the historiography of medical education. Funded by the Carnegie Foundation for the Advancement of Teaching, its author Abraham Flexner (1866–1959) proposed reforms in medical education in the United States and Canada: stricter admission requirements, standardised curricula leading to medical degrees and licences, a particular view of scientific medicine with the building of laboratories and schools of public health, and pre-medical courses in universities combined with clinical training in a hospital, along the lines of Johns Hopkins University. Two years later, in 1912, Flexner wrote another report on medical education in Europe. His recommendations were widely influential, facilitating massive investment from philanthropic organisations coupled with changes in curriculum and infrastructure to meet these new standards.^29^

Dozens of articles and books have assessed the impact of the *Flexner Report.* Although Lester S. King called it ‘probably the most grossly overrated document in American medical history’, Thomas Neville Bonner defended the report.^30^ In his trilogy on medical education in the United States, Kenneth M. Ludmerer interpreted the *Flexner Report* not as the beginning of educational reform in America, but as the result of cumulative efforts across the nation. In the first book of his trilogy, *Learning to Heal*, Ludmerer traced the rise of modern academic medicine alongside teaching hospitals and state licensure through a series of educational reforms since the nineteenth century in the United States. The second book in the trilogy, *Time to Heal*, discussed the changing nature of medical education in the twentieth century as health care in the United States became more privatised and for profit. This book is particularly valuable in highlighting the experiences of students and other members of medical schools in the United States. More recently, the third book in the trilogy, *Let Me Heal*, analysed the history of the internship system in the United States, uncovering the historical tension in hospitals between providing quality education and using the cheap labour of interns. Taken together, these three books show how today’s system of medical education has been shaped over at least two centuries of continuous change.^31^ Other scholars have focused more on the impact that the *Flexner Report* had on marginalised groups. Moya Bailey argued that the report implicitly promoted ‘northern wealthy white men’ as the ‘prototypical student’ in medicine, ultimately reinforcing dominant notions of gender, race, and regional difference. Similarly, Todd Savitt criticised how the report put pressure on ‘seven black medical schools,’ of which three eventually closed down.^32^ Several works have been published to assess the legacy of the Flexner model and how influential it can still be today, including to mark its centenary in 2010.^33^

Therefore, when it comes to the history of medical education in empires, it seems necessary to combine national and institutional histories by broadening the geographical scope to cover several metropoles and colonies, by modifying the methodology to stress comparisons and connections and by extending the chronology to span at least the past two centuries.

## Medical Education in Empires

This special issue draws on the historiography discussed here but emphasises medical education in the framework of empires. Colonial empires were built not only through military conquest, but also through political influence, imperial competition, commercial interests, resource exploitation and means of communication. They were driven by intangible forces such as racial ideologies and political theories. As empires expanded, standards and practises were promoted within them, including in medicine. Medical infrastructure, networks and sub-disciplines (e.g. tropical medicine, military medicine, parasitology, epidemiology) supported the emergence, expansion and consolidation of empires. However, the history of medicine in colonies has not been one of unidirectional diffusion, nor of coherent accumulation of knowledge. Empires competed with each other. Medical officials disagreed even within the same empire. Colonial staff and colonised populations clashed with imperial authorities. And the faith-based and private spheres (e.g. missionaries, charities, philanthropic institutions) did not always conform to government policies on medical practise.

To grasp the complexity of this everyday, flesh-and-blood life in empires, recent historiography has emphasised the pluralism, hybridity, exchange, negotiation and mutual transformation of medical practices and knowledge in colonial contexts. Guy N. A. Attewell, Kavita Sivaramakrishnan and Claudia Liebeskind, for example, have examined how the ancient Indian medical systems of Unani and Ayurveda were reshaped from the late nineteenth century onwards in a context of growing nationalism around expectations of training in institutions, publishing in academic journals and books, obtaining degrees, wearing certain clothes and using specific instruments.^34^ Karen E. Flint has traced how Zulu healers in Natal, in today’s South Africa, dealt with the category of ‘traditional’ healer, which could rigidify and place them in competition with British medicine, but which also became a means of gaining state recognition, when in fact medical practices were shaped by a range of influences from Europe, Africa and Asia, particularly India.^35^ In his study of Malawi at the turn of the twentieth century, Markku Hokkanen analysed how the coexistence of imperial doctors, Scottish missionaries and Africans created an environment of complex exchange, pluralism and power struggles in the name of the best ways to maintain health.^36^ Laurence Monnais also explored medical pluralism in colonial contexts, focusing on the territory of present-day Vietnam at the time of French colonial rule. She shows how, in practice, medicines from Europe and therapies from the colony could be combined in the form of hybrid remedies, as patients did not have exclusive loyalty to one form of treatment and the health market could thrive on the coexistence of multiple approaches.^37^ Sokhieng Au has analysed comparable dynamics in colonial Cambodia, a territory treated by some doctors as a laboratory, where pre-colonial and French medical systems coexisted, with the Khmers ultimately not so willing to rely on the medical programmes run by the French.^38^ In their recent edited volume on ‘the grey zones of medicine’, Diego Armus, Pablo F. Gomez and their colleagues explored the relationship between the institutionalised, official medicine of licensed practitioners that emerged in Latin America and the Caribbean between the seventeenth and twentieth centuries and the myriad types of unlicensed health practitioners. The result was a multifaceted medical environment emerging from the colonial era in which the experience of care was far from clear-cut boundaries between ‘traditional’ and ‘modern’ medicine.^39^ Another interesting case is China, informally influenced by several imperial powers, rather than being formally colonised by an empire. In the 1950s, after the Communist Revolution, efforts were made to ‘unify’ what was considered traditional Chinese and Western medicine, including through medical education.^40^ This is one of the themes that Steven Pieragastini and Martin Robert analyse in this issue, looking specifically at the case of Shanghai.

Our special issue differs from much of the historiography on medicine in colonial contexts in that it does not concentrate on the analysis of diseases, epidemics, pharmacopoeia or theories of the body and health, but on a more neglected aspect: medical education.^41^ It does so by focusing on empires, understood here in the broadest sense, including colonial, self-proclaimed, formal or informal empires—although the articles deal mainly with empires whose components were separated by oceans. The United States, for example, has never formally claimed to be an empire, but some of its strategies for influence beyond its national territory can be analysed within an imperial framework, as Christopher D. E. Willoughby does in this issue. It can also be argued—and some do—that settler colonies where there were indigenous populations and where territories were appropriated by settlers, or the dominions that launched their own colonial projects (a kind of ‘sub-empire’), have an imperial dimension to their history. Hohee Cho’s article discusses this aspect in relation to the Pacific Islands.

In the context of imperialism, medical education sometimes modified pre-colonial health traditions or fuelled national movements. For imperial authorities, training young people from the colonies in medicine could be a way of making them indebted to the Empire for their education, and gaining the sympathy of the local populations by providing health care. The flip side was that medical education could create a new educated local elite interested in politics, talking about autonomy and turning against the empire. Such fears were not entirely unfounded. Some medical schools in empires did serve as catalysts for independence movements and the building of national scientific cultures, as Hans Pols shows in this issue for the medical school founded by the Dutch in Batavia, now Jakarta. Other authors, such as Anne Ruddock and Pratik Chakrabarti in the case of India, Jethro Hernández Berrones in the case of Mexico and Marcel Rheault and Georges Aubin in the case of Quebec, have shown the predominant role played by doctors in movements for political affirmation against imperial powers or in post-colonial models of medicine and government.^42^ This kind of involvement by doctors was made possible through medical education, but also against it, when graduates criticised the education they received—Frantz Fanon being a well-known example.^43^

To some extent, the imperial authorities were aware of this risk of backfire when they set up medical schools in colonies. In 1912, for example, the French Minister of Foreign Affairs and the Minister of Colonies received a request from a student from Annam (a French protectorate in present-day Vietnam) for permission for him and his friends to travel to the French Faculty of Medicine in Beirut to complete their medical studies. Progress in medical education under the French system was limited in Annam, and these students did not have the means to study in Paris. The Minister of Foreign Affairs, Raymond Poincaré (later to become President of the French Republic), was in favour, in order to increase the influence of the French colonial institutions in Asia and to strengthen the links between them. But the request was rejected after the governor-general of Indochina argued that, in view of the recent political upheavals in Annam, it would be unwise for the French Empire to accept the request:

‘[o]ur Indo-Chinese protégés and subjects, as soon as they come into contact with modern civilisation, show a marked inclination to political discussion and abstract speculation, which makes it essential to control their actions closely and to monitor their intellectual development carefully. It is to be feared that in Beirut, left to themselves, deprived of guidance and direction, and mixed with an Ottoman and Levantine population, the young Indo-Chinese will soon become “uprooted” and gradually lose the feelings of gratitude and respect that should always bind them to France.’^44^

Although polysemic, the notion of civilisation referred to here is often associated with medical education in empires (sometimes explicitly as part of what is termed the ‘civilising mission’), with imperial authorities or their subordinates as gatekeepers. Similarly, Tim Livsey has shown that the British intentionally kept professional education (including medicine) at a subordinate level in Nigeria to maintain stratified power relations between the British and Nigerians.^45^

That medical education enabled students and graduates to negotiate with or defy empires means that medical education in empires cannot be understood solely as imposed by the colonisers on the colonised. An imperial power can have as much ambition to dominate medical activity in a colony as it wants. But if no one wants to enrol in its medical schools, and no one wants to be treated by the medical professionals it trains, those ambitions will quickly hit a wall.

The imperial authorities had to show prospective students what was in it for them, knowing that those who enrolled in a medical school had their own personal ambitions and could have collective projects. This is why, for example, some British or French medical schools in Asia offered scholarships to local students, the amount of which would increase with the number of years of study to discourage the students from dropping out, as examined in this issue for the cases of India and Indochina. In turn, imperial authorities could impose conditions on obtaining medical qualifications, such as the obligation to work in the medical service of the imperial army or in colonial medical institutions for a certain number of years after graduation.

What is clear from recent historiography, and from the articles collected in this issue, is that imperial contacts in medical education were characterised by negotiation, exchange, hybridisation and competition that shaped medical practices in their own ways and created multilayered networks of influence.^46^ To say that empires made hybrid medical universes possible is not to say that imperial medicine systematically created friendly cooperation in which everyone brought the best of their skills to the table. There were varying degrees of interaction, interest, disinterest and outright condemnation. For example, medical schools in early nineteenth-century Calcutta had curricula similar to those in Edinburgh, while also incorporating, at least for a time, elements of Indian Ayurvedic and Unani medicine. But there were other contexts in which the medical practices of colonised peoples were not taken into account at all, such as in Canada, where one searches in vain for the practices of indigenous peoples in official medicine during the same period, as shown by Martin Robert in this issue.^47^

Hence, there are irreducible differences between each colonial situation. This is why broad categories such as ‘indigenous’, ‘native’, ‘vernacular’ or ‘traditional’ medicine effectively allow access neither to the specificities nor the changes over time of the healing practices of peoples in colonies, even though such categories may have concrete implications, including for political affirmation in post-colonial contexts. Ideally, healing practices that have faced imperialism in recent centuries should not be lumped together in categories such as ‘vernacular’ (even if it’s not easy to find a more convenient vocabulary) if we are to understand what they are about.

Thus, empires and colonies were not monolithic. Neither were they isolated. They rubbed shoulders and shaped each other, comparing, sharing and trading practices and knowledge. As the article by Pieragastini and Robert in this issue shows, for example, the Hanoi medical school opened by the French at the beginning of the twentieth century was inspired by the French model of the Pondicherry medical school, but also by the models of the British medical schools established in India. Moreover, when the French imperial administration decided to support a medical school in Shanghai in the following years, it was because it wanted to compete with the rival imperial medical schools run by the Americans, British, Germans and Canadians in China.

Similarly, Hohee Cho’s article discusses how schools in the British Empire, such as those in Ceylon, Singapore or Uganda, influenced the curriculum of the medical school in Fiji, which attracted students from across the Pacific Islands. Other colonial medical schools also attracted students from various parts of the same empires or beyond. Those in Singapore and Uganda drew students from across British Malaya and British East Africa. Medical schools in Hong Kong and Singapore exchanged medical examiners.^48^ The schools in Fiji, Singapore, and Uganda were, therefore, regional hubs of medical education in the British Empire. This special issue opens avenues for exploring such dynamics between imperial powers and their associated colonies, in addition to inter-colonial and inter-imperial differences, cooperation and competition.

It also invites us to think about the feedback effect that medical education in colonies had on medical education in imperial metropoles, especially through travelling doctors, surgeons and students. Kirstin D. Hussey’s book about the repercussions of mobility of people within the British Empire on the medical landscape of late nineteenth-century and early twentieth-century London has recently yielded interesting results in this respect.^49^ Douglas M. Haynes’ book on the medical career of Patrick Manson (1844–1922) in China in relation to his later role as founder of the London School of Tropical Medicine also exemplifies such an approach.^50^ Historians Mark Harrison and Erica Charters, who have in many ways inspired this special issue, have written extensively on the multiple directions taken by trajectories and relationships of influence within empires in public health or military medicine, making crucial contributions in this field.^51^

With this in mind, three recent publications seem to us to best illustrate our special issue’s perspective on medical education in empires. The first is *Transforming Medical Education*, an edited volume by historians Delia Gavrus and Susan Lamb, published in 2022.^52^ This wide-ranging volume—reviewed by Martin Robert in this issue—does not specifically highlight the role of empires in medical education, but some of its chapters deal with the ways in which colonisation transformed medical education in different parts of the world, including Canada, a country that has hitherto been rather neglected on this subject. It is undoubtedly one of the most important syntheses of the history of medical education to be published in recent decades. Its chapters focus either on the period before 1750 or, for most of them, after 1880, that is, after the rise of bacteriology. In this issue, the articles by Martin Robert and Christopher D. E. Willoughby cover the intermediate period between the late eighteenth and late nineteenth centuries, offering an interesting complement to *Transforming Medical Education.*

Second, *Translating the Body: Medical Education in Southeast Asia*, published in 2017, examines the Dutch, British, French, Soviet and American imperial presence in what is now Indonesia, Malaysia, Vietnam, Laos, Cambodia, the Philippines and Thailand. The authors show how medical education conveyed a particular body culture and way of life, which they illustrate by looking at public health campaigns and how these promoted a sense of what the editors call ‘health citizenship’. Concentrating mainly on the twentieth century, the authors examine, among other aspects, how medical education played a key role in the creation of new national medical corps at the time of decolonisation.^53^ Our special issue builds on the perspectives offered by this book, not least because one of its editors, Hans Pols, contributes an article here, but also because Hohee Cho’s article presents innovative research on the functioning of medical education in the Pacific Islands, where several imperial authorities and private foreign organisations were involved in medical education.

Third, the book *Medical Education in East Asia: Past and Future* was published in 2017 to mark the centenary of the China Medical Board, a private foundation established by the Rockefeller Foundation and involved in health initiatives in East Asia. The book aims to celebrate the success of the health systems of China, Japan, South Korea, Taiwan and Hong Kong after a century of war, political unrest and poverty. Reflecting on the rise of East Asia over the past fifty years, the authors are explicitly forward looking, identifying the challenges that lie ahead to improve cooperation between these countries, promote the health of their populations and enable them to play a leading role in global medicine. The chapters in this book emphasise the importance of medical education in improving health, pointing out that health indicators and life expectancy are among the best in the world in East Asia, a region of independent nations whose history has been largely shaped by the British, French, Japanese, American, German and Dutch empires, as well as the USSR and private organisations such as the Rockefeller Foundation. Of particular interest is the insight the book provides into the relationship between the pre-twentieth-century world of empires, in which religious missionary congregations were largely involved in health care, and the medical world that has developed since the twentieth century around international organisations, private foundations and state health systems. In particular, it helps to understand how national medicine in East Asian countries has been built on both a ‘Western’ model of medicine and treatments recognised as traditional, pointing out that in ‘all East Asian countries today, traditional medicine coexists with Western medicine, enjoying both popular use and national support’, and that this is reflected in medical education.^54^ Exploring related themes, Pieragastini and Robert’s article herein allows for a critical reflection on the impact of empires in China because it deals with the case of French and Catholic medical education in Shanghai.

## Montreal, Calcutta, Boston, Batavia, Suva and Shanghai: Connecting the Dots

The five articles published in this issue draw on a variety of sources in languages including English, French, Dutch and Chinese to cover different periods (eighteenth to twentieth centuries), scales of analysis (city, region, sovereign state and continent), empires (British, Dutch, French, Japanese and the United States in its peculiar way), and non-state or quasi-state actors (British East India Company, Rockefeller Foundation and missionary congregations). The focus is on institutional medical education (hospitals, schools and faculties), but informal and private networks are also considered, including associations and interpersonal relationships. By comparing and linking cases that are not usually analysed together, the articles make it possible to trace the stages and differences in the life of medical schools in empires: who sponsored them and in what context; what models served as the basis for their creation; who taught in them and what they taught; who the students were, why they attended the schools and under what conditions; what the careers of graduates were like; and what social changes medical schools were involved in.

The issue opens with Martin Robert’s article on the first two colonies of the British Empire (excluding the United States) where colonial medical schools were established: Canada and India. To date, few studies have analysed the history of medicine in Canada and India together, despite the fact that they were linked by the British Empire during the same period. What we gain from a joint study of these two territories is that, for all their differences, there were striking similarities between their forms of medical education. From Calcutta to Montreal, British medical schools emphasised human dissection for the study of anatomy, clinical training at the bedside in hospitals and organic chemistry for the preparation of remedies.

No less revealing are the differences between the two contexts highlighted in Robert’s article. In India, the British East India Company established the first British medical schools in a top-down manner. These schools were designed to train subordinate Indian doctors to provide cheap medical labour for the British East India Company’s activities in a context where distance and difficult travel did not allow many European doctors to settle in India. The need for medical care increased because of wars and epidemics. British concerns about the effects of ‘hot climates’ on European bodies underpinned some of the teaching, particularly in anatomy. For a little more than a decade, Indian medical practices of Ayurveda and Unani were incorporated into teaching in these schools, with some British Orientalists seeing themselves as reviving ancient Indian medical traditions. This meant that the translation of medical treatises became an important part of the medical curriculum. The project of recruiting young Hindu and Muslim men into medical schools in India so that they would become public servants willing to treat any patient worked to some extent. But recruitment remained low and was not really boosted by scholarships, partly because the Indian population did not seem very interested in consulting British-trained doctors. In all cases, the intention from the outset was not just to treat imperial representatives. It was also to transform the medical and social world in India by creating a new class of professionals.

In Canada, however, the first medical schools were created from the bottom-up by local Protestant patricians. After the Revolution in the United States, Canada was the remnant of British North America—it was not high on the imperial metropole’s list of concerns. Early medical schools in Canada were designed primarily to provide a local option for young men interested in becoming doctors, rather than forcing them to go overseas, particularly to Britain or the United States. The United States in particular was a source of concern. The War of 1812 with the United States had exacerbated the conflict between loyalty to the British Crown, on the one hand, and republicanism on the other. In this context, as Rainer Baehre has shown, the idea of establishing medical schools in Canada was promoted by some as a way of discouraging Canadians from studying in the United States, where they would risk becoming republicans hostile to the British Empire. Unlike in India, Canada’s institutionalised medical education did not emphasise climate, nor did it include indigenous knowledge and care practises.

In short, Robert’s article shows how the British Empire created the conditions for a certain degree of standardisation in the training of medical students. However, this should not be seen as the beginning of a linear and unanimous process of standardisation of medical education. Rather, it was the beginning of an uneven and sometimes rough negotiation of the criteria of medical competence based on a range of colonial contexts. Some of the central themes of this article are echoed elsewhere in the special issue. One is the use of colonial medical schools to train second-class doctors who were imagined as intermediaries between the representatives of the empire and the local populations in the colonies, but who were tied to the colony by their titles of practise. Another is that medical education was more likely to be open to pre-colonial practices of health care when the metropole was distant and not easily accessible, making the colony function in a more isolated way within the empire, as in India.

Following the chronology, the next article in the issue is by Christopher D. E. Willoughby, on medical education in the United States before the American Civil War (1861–5). In the decades that followed the American Revolution, several medical schools began to appear in the United States. Willoughby emphasises that there existed a presumed hierarchy of human races in medical education, placing enslaved and indigenous people at the lowest rank. In doing so, part of medical teaching reinforced the idea that each climate corresponded to a particular body type, typically that dark-skinned bodies were adapted to warmer climates and pale-skinned bodies were adapted to colder climates. Based on this idea, doctors such as Daniel Drake made the medical argument that former slaves from the southern United States should move to Liberia on the African coast instead of the northern United States. Similarly, for a brief period, Harvard University’s medical school aimed to train free African Americans as doctors on the condition that they agree to settle in Liberia, another example of medical education that defined students’ professional horizons for imperial reasons. Further contributing to the essentialisation and naturalisation of racial categories, links between medicine and racial ideology were also found in student theses at the time, where race was used as a factor in analysing the differential impact of epidemics on bodies.

To show the connection between medical education on race in the United States and the history of empires, Willoughby draws both on the ways in which the activities of European imperialism were monitored in the United States, and on the imperial projects of the United States itself in different parts of the world. The author shows how imperially facilitated interpersonal and diplomatic networks allowed specimens from as far afield as British India to enter medical collections in the United States. Willoughby also shows how anatomists in the United States used data collected by doctors in colonial contexts to support their biological arguments for racial difference and hierarchy. Indirectly, textbooks and courses for medical students in the United States thus incorporated accounts of imperial situations, including wars from which human remains were taken, and epidemics. The government of the expanding United States itself, with its diverse indigenous peoples and climatic characteristics, could be seen as a micro-version of the imperial government of overseas colonies from different parts of the world.

It is not only the external impact of European empires on the United States that Willoughby is interested in. He also discusses how racial ideology, reinforced by imperial relations, was useful within the United States, which was then engaged in the westward colonisation of American territory to the detriment of indigenous peoples. Indigenous anatomical specimens soon appeared in the collections of medical schools, where their use included teaching what was then called racial science. The United States also launched expeditions to gain influence and control beyond its borders, particularly in the Caribbean, the Pacific and in what became Liberia in Africa. These ventures, motivated to some extent by imperial intentions, were underpinned by medical doctrines of racial hierarchy, which in turn were reinforced by new opportunities to observe, compare and collect data and specimens. Taken together, Robert’s and Willoughby’s articles suggest the constitution of a North American world of medical education shaped by the conflicts and trades in which European empires were involved.^55^

In the next article, Hans Pols, describes how institutionalised medical education had modest beginnings in the capital of the former Dutch East Indies, Batavia, now Jakarta, in Indonesia. In the twentieth century, however, medical education in Batavia would become a springboard for nationalist movements at the time of Indonesian independence (1945–9).

The story begins in the mid-nineteenth century. Following a devastating epidemic, Batavia’s first medical school was established by the Dutch imperial administration. The intention was not to train medical minds, but to train more sets of medical hands. Providing practical training for Indonesians to meet the specific needs of the colony and prevent further health crises was the aim of the medical school in Batavia. The imperial administration attracted prospective students by offering scholarships, as in other cases analysed in this issue. However, these scholarships were conditional on working for colonial services after graduation.

Pols shows that once training began at the school, a dynamic ensued whereby Indonesian medical graduates took on the role of a new educated middle class. They became more prominent in their communities and expected to be valued for their skills and social usefulness. Following a pattern seen in other cases collected in this issue, these Indonesian doctors became an ambiguous ‘subaltern elite’, occupying an uneasy position between the European representatives of the Empire and the population that did not have their level of education.^56^ Some medical students adopted a Dutch lifestyle (in dress, diet, political views, etc.), inspired by the ideals of progress, modernity, scientific knowledge and the opportunities promised by learning Dutch. At the same time, there was contempt for their ambitions on the part of Europeans, who acted in a way that showed Indonesian doctors that they should remain on the fringes of the Empire and the professional world. Nevertheless, in practise, medical education gave Indonesian doctors greater social responsibility. It also created an environment in which they could meet and organise themselves into student and scientific associations.

The stage was set for the new Indonesian medical elite to clearly see what they rejected in the imperial administration. Unable to obtain the recognition and responsibility for their profession that they had imagined they would obtain in the Dutch Empire, many of them concluded that they had to get rid of imperial rule. This way, Indonesian doctors, through their common education, were instrumental in the independence of Indonesia. An important moment in this history was the Second World War, during which Batavia was occupied by Japan. This followed decades of investment in medicine in the region by an American philanthropic organisation, the Rockefeller Foundation. For Pols, these actors represented alternatives that Indonesian doctors could look to as proof that models of modernity different from those in continental Europe were possible. The idea of an Indonesian nation whose health should be looked after by Indonesian doctors thus emerged after the war, accelerating the fall of the Dutch imperial regime. This is an example of how an empire’s medical school, originally established for practical reasons, was reappropriated by its students as a means of fighting imperial rule.

From Batavia, we head to the Pacific for the next article by Hohee Cho on the Central Medical School in Fiji. This research is particularly original as there is very little work on the history of medicine in the Pacific archipelagos. Certain islands there fell under the British Empire in various configurations, others under the French Empire. There were even condominiums whose administration was shared by two empires, not to mention the islands administered by Australia or New Zealand, a kind of sub-imperial status, in addition to the role played by the United States and, again, by the Rockefeller Foundation during the twentieth century. In short, the world of the Pacific Islands was a highly complex one, made up of small and often sparsely populated territories far apart from each other, where the overlap and combination of jurisdictions were the norm, especially for the empires of the time.

The reason why a medical school was first created in this context was, as in the case of Pols’ article, a devastating epidemic that threatened to decimate the population of certain islands. Largely for economic reasons (a healthy workforce), but also with reference to the anthropological preservation of human races, representatives of imperial administrations along with the Rockefeller Foundation launched a project to establish one medical school for multiple colonies. This was to become the Central Medical School, located in Fiji, the first version of which opened its doors in 1885. This is an extreme case of overlapping empires within a single institution. As Cho puts it, quoting an archive document, the Central Medical School became a ‘microcosm of the Pacific’. A quota system was introduced to determine the number of students from each island. Sponsored by the Rockefeller Foundation, the school was run by the colonial government of Fiji, staffed by British tutors, and jointly administered by the colonial administrations of Britain, Australia, New Zealand, France and the United States. It enrolled students from twelve distinct territories.

Scholarships were offered by the school administration. Although the school’s rules were designed to insulate the students in their respective cultures, particularly through clothing regulations, Cho notes that some of the students used their allowance to buy European clothes or objects. Rituals such as the graduation ceremony, at which students wore traditional-style uniforms while the High Commissioner for the Western Pacific wore a British gown, reinforced the idea that the school was producing subordinate doctors who were exposed to European ways of life and encouraged to admire them, even if they had to remain on their islands.

The Central Medical School in Fiji had the effect of officialising the prejudices held by imperial administrators about Pacific peoples, such as the supposed similarities between the Pacific Islanders in their adaptiveness to island environments and the presumed differences in ability between Melanesians and Polynesians. Here, too, racial hierarchies were part of medical education, albeit in a different way than in the case analysed by Willoughby. Because of the multi-ethnic nature of the Fiji school, students from different islands became representatives of the population from which they came.

The final article, by Steven Pieragastini and Martin Robert, looks at China, and more specifically at the French concession in Shanghai in the first half of the twentieth century. This case is also unusual, but in yet another way. This was not a colony in the strict sense, but a city partly under French administration because of a concession (1849–1943). The French Concession coexisted in Shanghai with the presence of other foreign powers, including the United States, the United Kingdom and Germany. But it was not just foreign civil powers that were there. Missionary orders, notably the Jesuits, had been well established in Shanghai since the nineteenth century. By the early twentieth century, some of the activities of these religious orders were intertwined with those of the civil authorities in the concessions, including through educational institutions. Pieragastini and Robert analyse how, in this context, a faculty of medicine existed in the French concession of Shanghai between 1912 and 1952 and how it became a means for both France and Catholic representatives to consolidate their influence throughout Asia and beyond. The authors argue that the key to the faculty’s impact was that it was able to operate with the support of both the Jesuits and French diplomacy, without presenting itself as either a missionary or an imperial institution.

The article considers the French medical schools established in Asia, including in Pondicherry (1863) and Hanoi (1902), but also in southern China. An analysis of French colonial archives shows that letters and personnel circulated between these schools, demonstrating that French medical education in Asia was conceived not only as a way of meeting the needs of the colonies, but also more broadly as a way of establishing French imperial authority on the Asian continent. The rationale was that by training young locals in medicine, they would be exposed to the French way of life and become indebted to France for their education and social status. In addition, the population receiving care from these local doctors would be more sympathetic to the imperial authority providing these medical services. French medical schools in Asia, like most others analysed in this special issue, were not usually designed to train erudite scientists, but to provide basic practical training. In this respect, empires drew inspiration from each other for their medical schools.

It was against this backdrop that the faculty of medicine was established in the French Concession in Shanghai in 1912. This institution was intended to strengthen the French influence in Asia, in cooperation with similar institutions, particularly those in Indochina. However, the Shanghai Faculty was unusual in several respects. In association with a French hospital, it was an addition to a university founded by the Jesuits, *l’Aurore* (Aurora University). The faculty was therefore not just a practical school. It was part of a university. Those who enrolled, mostly Chinese, but also many Russians and other foreigners, became full-fledged doctors. Many of them went on to complete their studies and work abroad, including in France.

The faculty of medicine of *l’Aurore* managed to become an important centre of French influence in Asia, as a result of the collaboration between the Jesuits and the anti-clerical French Third Republic, and despite the fact that it was a project of imperial influence at a time when the regime in China was republican and sought to supervise foreign institutions on its territory. Its students and graduates forged strong links with each other and maintained their networks through publications, associations, and even marriages between former students. To avoid antagonising the Chinese authorities, the faculty presented itself as a strictly scientific institution, offering an elite and cosmopolitan education and opening the horizons of its graduates. Many graduates took up positions in health care institutions throughout China, often in hospitals run by religious communities, as well as abroad.

Still, the ‘medical ethics’ courses, taught by a Jesuit Father, were a way to exercise religious influence at the faculty. Ethical principles based on Christian teachings formed the basis of the oath that the medical students took upon graduation. An important part of the teaching of medical ethics focused on birth control. Most members of the faculty were opposed to birth control and abortion on Christian grounds. At the same time, they opposed, also on Christian grounds, the eugenic policies that were gaining popularity in Europe in the 1930s.

The article concludes by examining what happened to the faculty during the convergence of the Second Sino-Japanese War (1937–45) and the Second World War (1939–45). A large proportion of the French medical staff left China during this period. As a result, Chinese staff played a greater role in the Faculty, in which interest in traditional Chinese medicine was growing. A Pasteur Institute opened in Shanghai, working closely with the faculty. After the wars and the proclamation of the People’s Republic of China (1949), the university came under severe criticism for its links with foreign empires and religious communities. The faculty of medicine was forced to merge with other institutions to form a new medical school. What is striking is that several doctors who had been trained at *l’Aurore* continued to practise under the communist regime because of public health needs and the lack of alternatives. Pieragastini and Robert thus suggest that the role of medical schools in empires cannot be understood solely in terms of formal colonies. Concessions, among other forms of imperial settings, were also important in medical networks of influence. This article is equally a reminder that religious missionary communities must be taken into account when studying medicine in empires.

Together, the authors in this special issue call for greater attention to the connections that have existed within and across empires through medical education. Formal empires may have largely disappeared today. But the graduation ceremonies of many medical schools still reflect the global connections in medical education, not entirely unlike the graduation of the first seven Koreans to receive medical licences in June 1908. Students train in multi-racial cohorts, take the Hippocratic Oath and receive qualifications that can be recognised nationally and even internationally. Graduates then practise medicine in their countries or abroad following constant negotiations and conflicts between regulatory bodies to determine whether and under what conditions a doctor is considered useful, safe and competent. The international movement of medical personnel today largely follows linguistic and historical ties between different regions of the world that were once part of the same empires, such as between South Asia and the United Kingdom, or North Africa and France. This special issue aims to reaffirm the relevance of medical history for historians of empires, and the significance of the history of empires for historians of medicine. Hopefully, it will also serve to highlight the importance of historical perspectives in medicine more generally, including in the education of future health professionals.^57^

## Footnotes

1 The editors thank all of the colleagues whose indispensable support made this special issue possible. Our gratitude goes first to the University of Oxford’s Centre for the History of Science, Medicine, and Technology for hosting the workshop held on 21–22 June 2021, from which this issue emerged. We also thank the discussants and participants in the workshop panels and our colleagues whose thoughtful comments were essential in bringing the articles presented here to their final form, namely: Evan Bonney, Erica Charters, Belinda Clark, Tolulope Esther Fadeyi, Ethan Friederich, Mark Harrison, Hoi-eun Kim, Jacqueline Knörr, Jagyoseni Mandal and Wayne Soon. We are grateful for the support of the editors of *Medical History*, especially Tara Alberts, Sanjoy Bhattacharya and Akihito Suzuki, as well as all the anonymous reviewers who generously offered their comments on the articles. The preparation of this article was supported by the Social Sciences and Humanities Research Council of Canada, the *Fonds québécois de recherche – Société et culture*, and the *Institut francilien recherche, innovation, société.*

2
*The Korea Mission Field,* 4, 8 (1908), 123–127; Hyoung Woo Park, Yun Jae Park, In-Sok Yeo and Il Soon Kim, ‘Medical Education in Je Joong Won’, *Korean Journal of Medical History*, 8, 1 (1999), 33–55; https://newsteacher.chosun.com/site/data/html_dir/2023/10/18/2023101803248.html.

3 Tony Ballantyne, *Webs of Empire: Locating New Zealand’s Colonial Past* (Vancouver: UBC Press, 2014). See also: Jean-Paul Zuñiga, *Constellations d’empire. Territorialisation et construction impériale dans les Amériques hispaniques (XVIIe-XVIIIe siècle)* (Madrid: Casa de Velázquez, 2023), which develops the notion of imperial “constellations”.

4 For a synthesis of the way in which the world was becoming both more uniform in certain respects, such as clothing, and more prone to various manifestations of specificity that sometimes reacted to this uniformisation, see Christopher Bayly, *The Birth of the Modern World, 1780–1914* (Hoboken: Wiley-Blackwell, 2004).

5 On licensing and medical education, see Ruth Horowitz, *In the Public Interest: Medical Licensing and the Disciplinary Process* (New Brunswick: Rutgers University Press, 2013), 32–56.

6 It would be impossible to compile this vast literature here. A quick search of any university library catalogue will provide many examples. Here are a few examples not mentioned elsewhere in this article: Sunil Pandya, *Medical Education in Western India. Grant Medical College and Sir Jamsetjee Jejeebhoy’s Hospital* (Newcastle upon Tyne: Cambridge Scholars Publishing, 2019); Laura Kelly, *Irish Medical Education and Student Culture, c.1850*–*1950* (Liverpool: Liverpool University Press, 2017); Philip Rieder, *Anatomie d’une institution médicale. La Faculté de médecine de Genève (1876-1920)* (Genève: Éditions BHMS, 2009); Charles Coury, *L’enseignement de la médecine en France, des origines à nos jours* (Paris: Expansion Scientifique Française, 1968).

7 On the United States and the United Kingdom, see for example: Antonio M. Gotto, Jennifer Moon and Laurie H. Glimcher, *Weill Cornell Medicine: A History of Cornell’s Medical School* (Ithaca: Cornell University Press, 2016); Alun Roberts, *The Welsh National School of Medicine, 1893–1931: the Cardiff Years* (Cardiff: University of Wales Press, 2008); Keir Waddington, *Medical Education at St Bartholomew’s Hospital, 1123*–*1995* (Woodbridge, Suffolk: Boydell Press, 2003); Gerard N. Burrow, *A History of Yale’s School of Medicine: Passing Torches to Others* (New Haven: Yale University Press, 2002); Johanna Geyer-Kordesch and Fiona A Macdonald, *The History of the Royal College of Physicians and Surgeons of Glasgow, 1599–1858: Physicians and Surgeons in Glasgow* (London: Hambledon Press, 1999), combined with Andrew Hull and Johanna Geyer-Kordesch, *The History of the Royal College of Physicians and Surgeons of Glasgow: The Shaping of the Medical Profession* (London: Hambledon Press, 1999); Roy Porter and Vivian Nutton, *The History of Medical Education in Britain* (Leiden: Brill, 1995); Charles Newman, *The Evolution of Medical Education in the Nineteenth Century* (Oxford: Oxford University Press, 1957). On the Royal College of Physicians of London: George Clark, *A History of the Royal College of Physicians of London, Volume I* (Oxford: Clarendon Press, 1964); George Clark, *A History of the Royal College of Physicians of London, Volume II* (Oxford: Clarendon Press, 1966); A. M. Cooke, *A History of the Royal College of Physicians of London, Volume III* (Oxford: Clarendon Press, 1972); Asa Briggs, *A History of the Royal College of Physicians of London, Volume IV* (Oxford: Oxford University Press, 2005). Ludmerer’s work will be discussed below.

8 Useful resources on this topic include: Rafael Mandressi, *Le Regard de l’anatomiste. Dissections et invention du corps en Occident* (Paris: Seuil, 2003); Othmar Keel, *L’avènement de la médecine clinique moderne en Europe, 1750-1815: politiques, institutions et savoirs* (Montreal: Presses de l’Université de Montréal, 2001); Guenter B. Risse, ‘La synthèse entre l’anatomie et la clinique’ in M. Grmek (ed.), *Histoire de la pensée médicale en Occident, tome 2* (Paris: Seuil, 1997), 177–97; Michel Foucault, *Naissance de la clinique: une archéologie du regard médical* (Paris: Presses Universitaires de France, 2015 [1963]); Ronald L. Numbers (ed.), *The Education of American Physicians: Historical Essays* (Berkeley: University of California Press, 1980). See also: Martin Robert, *Cette science nécessaire. Dissections humaines et formation médicale au Québec* (Montreal and Kingston: McGill-Queen’s University Press, 2023).

9 George Basalla, ‘The Spread of Western Science’, *Science*, 156 (1967), 611–22; Warwick Anderson, ‘Remembering the Spread of Western Science’, *Historical Records of Australian Science*, 29, 2 (2018), 73–81.

10 W. F. Bynum, *Science and the Practice of Medicine in the Nineteenth Century* (Cambridge: Cambridge University Press, 1994).

11 W. F. Bynum, ‘The Rise of Science in Medicine, 1850–1913’ in W. F. Bynum, Anne Hardy, Stephen Jacyna, Christopher Lawrence and E. M. Tansey (eds), *The Western Medical Tradition, 1800 to 2000* (Cambridge: Cambridge University Press, 2006), 135–50.

12 Othmar Keel, ‘The Politics of Health and the Institutionalisation of Clinical Practices in Europe in the Second Half of the Eighteenth Century’ in W. F. Bynum and Roy Porter (eds), *William Hunter and the Eighteenth-Century Medical World* (Cambridge: Cambridge University Press, 1985), 207–256. He develops this point in his book: Keel, op. cit. (note 8).

13 Thomas Neville Bonner, *Becoming a Physician: Medical Education in Great Britain, France, Germany, and the United States* (Oxford: Oxford University Press, 1996). George Weisz’s book on the rise of medical specialties is also an example of an analysis of medicine that takes a comparative approach to different nations (France, the United Kingdom, the German-speaking world and the United States), although it is not primarily concerned with medical education: George Weisz, *Divide and Conquer. A Comparative History of Medical Specialization* (Oxford: Oxford University Press, 2006).

14 Charles Donald O’Malley (ed.), *The History of Medical Education: An International Symposium held February 5–9, 1968* (Berkeley: University of California Press, 1970). The few comparable examples are: the collective volume edited by Delia Gavrus and Susan Lamb, which we discuss later; Teizo Ogawa (ed.), *History of Medical Education: Proceedings of the 6th International Symposium on the Comparative History of Medicine — East and West, September 17–24, 1981, Susono-shi, Shizuoka, Japan* (Tokyo: Taniguchi Foundation Division of Medical History, 1983); Theodor Puschmann, *A History of Medical Education from the Most Remote to the Most Recent Times* (London: H. K. Lewis, 1891), originally published in German in 1889. See also: John F. Fulton ‘History of Medical Education’, *The British Medical Journal*, 2, 4834 (1953), 457–61.

15 Ole Peter Grell, Andrew Cunningham, and Jon Arrizabagala (eds), *Centres of Medical Excellence? Medical Travel and Education in Europe, 1500–1789* (Farnham: Ashgate, 2010).

16 See, for instance: Lindsey Fitzharris, *The Butchering Art. Joseph Lister’s Quest to Transform the Grisly World of Victorian Medicine* (New York: Farrar, Straus and Giroux, 2017).

17 Erwin H. Ackerknecht, *Medicine at the Paris Hospital, 1794-1848* (Baltimore: John Hopkins University Press, 1967); George Weisz, ‘The Politics of Medical Professionalization in France, 1845-1848’, *Journal of Social History*, 12, 1 (1978), 3–30; George Weisz, ‘Reform and Conflict in French Medical Education, 1870-1914’, in Robert Fox and George Weisz (eds), *The Organization of Science and Technology in France, 1808–1914* (Cambridge: Cambridge University Press, 1980), 61–94; Toby Gelfand, *Professionalizing Modern Medicine. Paris Surgeons and Medical Science and Institutions in the 18^th^ Century* (Westport: Greenwood Press, 1980); Lisa Rosner, *Medical Education in the Age of Improvement: Edinburgh Students and Apprentices, 1760*–*1826* (Edinburgh: Edinburgh University Press, 1991); John Harley Warner, *Against the Spirit of System: The French Impulse in Nineteenth-Century American Medicine* (Princeton: Princeton University Press, 1998); Florent Palluault, ‘Medical Students in England and France, 1815–1858: A Comparative Study’ (unpublished DPhil thesis: University of Oxford, 2003); M. Anne Crowther and Marguerite W. Dupree, *Medical Lives in the Age of Surgical Revolution* (Cambridge: Cambridge University Press, 2007); Aro Velmet, *Pasteur’s Empire: Politics and Bacteriology in France and its Colonies, 1890–1940* (Oxford: Oxford University Press, 2020); Matheus Alves Duarte da Silva, ‘Quand la peste connectait le monde: production et circulation de savoirs microbiologiques entre Brésil, Inde et France (1894–1922)’ (unpublished PhD thesis: EHESS, 2020). See also: Martin Robert, ‘Voyager pour apprendre: les Canadiens reçus docteurs en médecine à Paris au XIX^e^ siècle’, *Canadian Bulletin of Medical History/Bulletin canadien d’histoire de la médecine*, 39, 1 (2022), 72–98.

18 Bynum, *op. cit.* (note 11), 141.

19 Godelieve van Heteren, ‘Students Facing Boundaries: The Shift of Nineteenth-Century British Student Travel to German Universities and the Flexible Boundaries of a Medical Educational System’ in Nutton and Porter, *op. cit.* (note 7), 294.

20 Hoi-eun Kim, *Doctors of Empire: Medical and Cultural Encounters Between Imperial Germany and Meiji Japan* (Toronto: University of Toronto Press, 2014).

21 Hull and Geyer-Kordesch, *op. cit.* (note 7), 92–4; J. Rostowski, ‘Polish School of Medicine. University of Edinburgh, 1941–49’, *British Medical Journal*, 1 (28 May 1966), 1349–51.

22 Laura Kelly, *Irish Women in Medicine, c. 1880s–1920s. Origins, Education and Careers* (Manchester: Manchester University Press, 2012); Susan Wells, *Out of the Dead House: Nineteenth-Century Women Physicians and the Writing of Medicine* (Madison: University of Wisconsin Press, 2001); Tracey L. Adams, *A Dentist and a Gentleman: Gender and the Rise of Dentistry in Ontario* (Toronto: University of Toronto Press, 2000).

23 Douglas M. Haynes, *Fit to Practice: Empire, Race, Gender and the Making of British Medicine, 1850–1980* (Rochester: University of Rochester Press, 2017).

24 Sasha Mullaly and David Wright, *Foreign Practices. Immigrant Doctors and the History of Canadian Medicare* (Montreal and Kingston: McGill-Queen’s University Press, 2020).

25 Rebecca Martin, ‘Normalising Whiteness: the Use of Standardised Anatomical Models in British University Teaching, 1860–1910’ (unpublished PhD thesis, University College London, 2020).

26 Including: Greta Jones, *‘Doctors for Export’. Medical Migration from Ireland c. 1860 to 1960* (Leiden: Brill, 2021); Fallon Kiran Mody, ‘Doctors Down Under: European Medical Migrants in Victoria (Australia), 1930–60’ (unpublished PhD thesis: University of Melbourne, 2018); Julian M. Simpson, *Migrant Architects of the NHS. South Asian Doctors and the Reinvention of British General Practice (1940s–1980s)* (Manchester: Manchester University Press, 2018); Kieran Fitzpatrick, ‘Tense Networks: Exploring Medical Professionalization, Career Making and Practice in an Age of Global Empire, Through the Lives and Careers of Irish Surgeons in the Indian Medical Service, c. 1850–1920’ (unpublished DPhil thesis, University of Oxford, 2016); Laurence Monnais and David Wright (eds), *Doctors Beyond Borders: The Transnational Migration of Physicians in the Twentieth Century* (Toronto: University of Toronto Press, 2016); Anna Greenwood and Harshad Topilawa, *Indian Doctors in Kenya, 1895–1940. The Forgotten History* (London: Palgrave Macmillan, 2015); Rupalee Verma, ‘Western Medicine, Indigenous Doctors and Colonial Medical Education. A Case for Desire of “Hegemony” in Conflict with Demands of “Colonial Partiality”’, *Itinerario*, 19, 3 (1995), 130–41.

27 ‘Summary of the Medical Teaching Schools in the Colonial Empire’, *The National Archives at Kew* (TNA): CO83/235/13.

28 Douglas M. Haynes, *op. cit.* (note 23), 36–55.

29 See for instance: Marianne P. Fedunkiw, *Rockefeller Foundation Funding and Medical Education in Toronto, Montreal and Halifax* (Montreal and Kingston: McGill-Queen’s University Press, 2005).

30 Lester S. King, ‘The Flexner Report of 1910’, *JAMA*, 251, 8 (1984), 1079–86. Thomas Neville Bonner, ‘Abraham Flexner and the Historians: Guest Editorial’, *The Journal of the History of Medicine and Allied Sciences,* 45/1 (1990) 3–10.

31 Kenneth M. Ludmerer, *Let Me Heal: The Opportunity to Preserve Excellence in American Medicine* (Oxford: Oxford University Press, 2014); *Time to Heal: American Medical Education from the Turn of the Century to the Era of Managed Care* (Oxford: Oxford University Press, 1999); *Learning to Heal: The Development of American Medical Education* (Baltimore: Johns Hopkins University Press, 1985).

32 Moya Bailey, ‘The Flexner Report: Standardizing Medical Students Through Region-, Gender-, and Race-Based Hierarchies’, *American Journal of Law & Medicine*, 43 (2017), 209; Todd Savitt, ‘Abraham Flexner and the Black Medical Schools’, *Journal of the National Medical Association*, 98, 9 (2006), 1420.

33 See for instance: Thomas P. Duffy, ‘The Flexner Report–100 Years Later’, *Yale Journal of Biology and Medicine,* 84 (2011), 269–76; Warren D. Anderson, ‘Outside Looking In: Observations on Medical Education Since the Flexner Report’, *Medical Education*, 45 (2011), 29–35; Mike Mitka, ‘The Flexner Report at the Century Mark: A Wake-up Call for Reforming Medical Education’, *JAMA*, 303, 15 (2010), 1465–66; Donald A. Barr, *Questioning the Premedical Paradigm: Enhancing Diversity in the Medical Profession a Century after the Flexner Report* (Baltimore: Johns Hopkins University Press, 2010); Kenneth M. Ludmerer, ‘Commentary: Understanding the Flexner Report’, *Academic Medicine*, 85, 2 (2010), 193–96; Lawrence Diller, ‘100 Years later, the Flexner Report is Still Relevant’, *The Hastings Center Report,* 40, 5 (2010), 3; Molly Cooke *et al.*, ‘American Medical Education 100 Years After the Flexner Report’, *The New England Journal of Medicine*, 355, 13 (2006), 1339–44; Barbara M. Barzansky and Norman Gevitz (eds), *Beyond Flexner: Medical Education in the Twentieth Century* (New York: Greenwood Publishing Group, 1992).

34 Guy N. A. Attewell, *Refiguring Unani Tibb. Plural Healing in Late Colonial India* (Hyderabad: Orient Blackswan, 2018); Kavita Sivaramakrishnan, *Old Potions, New Bottles. Recasting Indigenous Medicine in Colonial Punjab, 1850–1945* (Hyderabad: Orient Longman, 2006); Claudia Liebeskind, ‘Arguing science. Unani Tibb, Hakims, and Biomedicine in India, 1900-50’ in Waltraud Ernst (ed.), *Plural Medicine, Tradition and Modernity, 1800–2000* (London: Routledge, 2002), 58–75 (see also the other chapters in this collective volume). For a similar dynamic in the context of nineteenth century Iran, see Hormoz Ebrahimnejad, ‘The Waqf, the State and Medical Education in Nineteenth-Century Iran’ in H. Ebrahimnejad (ed.), *The Development of Modern Medicine in Non-Western Countries. Historical Perspectives* (London: Routledge, 2008), 59–81.

35 Karen E. Flint, *Healing Traditions: African Medicine, Cultural Exchange, and Competition in South Africa, 1820-1948* (Athens: Ohio University Press, 2008).

36 Markku Hokkanen, *Quest for Health in Colonial Society. Scottish Missionaries and Medical Culture in the Northern Malawi Region, 1875–1930* (PhD thesis, University of Jyväskylä, 2006). See also: Markku Hokkanen, *Medicine, Mobility and the Empire. Nyasaland Networks, 1859–1960* (Manchester: Manchester University Press, 2017) and Markku Hokkanen and Kalle Kananoja (eds), *Healers and Empires in Global History: Healing as Hybrid and Contested Knowledge* (London: Palgrave MacMillan, 2019).

37 Laurence Monnais, *The Colonial Life of Pharmaceuticals. Medicine and Modernity in Vietnam* (Cambridge: Cambridge University Press, 2019). See also: Monnais-Rousselot, ‘Ordonnance coloniale, prescriptions médicales et changement social. L’offre et la demande en médicaments dans le Viêt Nam de la première moitié du XX^e^ siècle’, *Genèses*, 4, 69 (2007), 26–48.

38 Sokhieng Au, *Mixed Medicines. Health and Culture in French Colonial Cambodia* (Chicago: University of Chicago Press, 2011).

39 Diego Armus and Pablo F. Gómez (eds), *The Gray Zones of Medicine. Healers & History in Latin America* (Pittsburgh: University of Pittsburgh Press, 2021).

40 Xiaoping Fang, ‘Communist Medicine: The Emergence of TCM and Barefoot Doctors, Leading to Contemporary Medical Markets’ in Vivienne Lo and Michael Stanley-Baker (eds), *Routledge Handbook of Chinese Medicine* (London: Routledge, 2015), 638–648; Bridie Andrews, *The Making of Modern Medicine in China, 1850-1960* (Vancouver: UBC Press, 2014). See also: Mary Augusta Brazelton, ‘Western Medical Education on Trial: The Endurance of Peking Union Medical College, 1949–1985’, *Twentieth-Century China*, 40, 2 (2015), 126–45; Kim Taylor, *Chinese Medicine in Early Communist China, 1945–1963. A Medicine of Revolution* (London: Routledge, 2005).

41 Other examples include: Suman Seth, *Difference and Disease: Medicine, Race, and the Eighteenth-Century British Empire* (Cambridge: Cambridge University Press, 2018); Hans Pols, *Nurturing Indonesia: Medicine and Decolonisation in the Dutch East Indies* (Cambridge: Cambridge University Press, 2018) – which does, however, touch on medical education; Guillaume Lachenal, *Le médecin qui voulut être roi. Sur les traces d’une utopie coloniale* (Paris: Seuil, 2017); Samir Boumediene, *La colonisation du savoir: une histoire des plantes médicinales du “Nouveau Monde” (1492–1750)* (Vaulx-en-Velin: Édition des Mondes à Faire, 2016); Laurence Monnais and Hans Pols, ‘Health and Disease in the Colonies: Medicine in the Age of Empire’ in Robert Aldrich and Kirsten McKenzie (eds), *The Routledge History of Western Empires* (Abingdon-on-Thames: Routledge, 2014), 270–84; Pratik Chakrabarti, *Medicine and Empire: 1600–1960* (London: Palgrave Macmillan, 2013); Projit Bihari Mukharji, *Nationalizing the Body: The Medical Market, Print, and Daktari Medicine* (London: Anthem Press, 2009); David Arnold, *Colonizing the Body: State Medicine and Epidemic Disease in Nineteenth-Century India* (Berkeley: University of California Press, 1993). Some of the chapters in this collective volume deal with medical education: Poonam Bala (ed.), *Contesting Colonial Authority: Medicine and Indigenous Responses in Nineteenth- and Twentieth-Century India* (Lanham: Lexington Books, 2012).

42 Anne Ruddock and Pratik Chakrabarti, ‘The “Indian Predicament”: Medical Education and the Nation in India, 1880–1956’ and Jethro Hernández Berrones, ‘An Undesirable Past: Free Medical Schools and the First Doctors of the Mexican Revolution, 1910–45’ in Delia Gavrus and Susan Lamb (eds), *Transforming Medical Education: Historical Case Studies of Teaching, Learning, and Belonging in Medicine in Honour of Jacalyn Duffin* (Montreal and Kingston: McGill-Queen’s University Press, 2022), respectively 163–86 and 208–32; Marcel J. Rheault and Georges Aubin, *Médecins et patriotes. 1837–1838* (Quebec City: Septentrion, 2006). See also: Mody Kanté, *L’école de médecine de Dakar. Creuset de la formation d’une élite médicale africaine* (Paris: L’Harmattan, 2023).

43 Frantz Fanon, *Peau noire, masques blancs* (Paris: Seuil, 1952). See also: Delphine Peiretti-Courtis, *Corps noirs et médecins blancs. La fabrique du préjugé racial, XIX^e^-XX^e^ siècles* (Paris: La Découverte, 2021).

44
*Archives nationales d’outre-mer*, Aix-en-Provence, GGI 2680, September 1912. Martin Robert’s translation from French.

45 Tim Livsey, *Nigeria’s University Age: Reframing Decolonisation and Development* (London: Palgrave Macmillan, 2017), 19–40.

46 In this respect, critiquing George Basalla’s article, *op. cit.* (note 9) became an important part of teaching and research in the history of science. On this historiographical transition, see for example: Alan Lester, ‘Imperial Circuits and Networks: Geographies of the British Empire’, *History Compass*, 4, 1 (2006), 124–41; Mark Harrison, ‘A Global Perspective: Reframing the History of Health, Medicine, and Disease’, *Bulletin of the History of Medicine*, 89, 4 (2015), 639–89.

47 On this topic, see also: Maureen Lux, *Separate Beds: A History of Indian Hospitals in Canada*, 1920–1980 (Toronto: University of Toronto Press, 2016); Maureen Lux, *Medicine that Walks. Disease, Medicine, and Canadian Plains Native People, 1880–1940* (Toronto: University of Toronto Press, 2001).

48 ‘Summary of the Medical Teaching Schools in the Colonial Empire’, TNA: CO83/235/13.

49 Kirstin D. Hussey, *Imperial Bodies in London. Empire, Mobility, and the Making of British Medicine, 1889-1914* (Pittsburgh: University of Pittsburgh Press, 2021). On the historiographical context that has led historians to take an interest in the ways in which empires have shaped Europe, see Richard Drayton, ‘Science, Medicine, and the British Empire’ in Robin Winks and William Roger Louis (eds), *The Oxford History of the British Empire, vol. V: Historiography* (Oxford: Oxford University Press, 1999), 264–76.

50 Douglas M. Haynes, *Imperial Medicine: Patrick Manson and the Conquest of Tropical Disease* (Philadelphia: University of Pennsylvania Press, 2013).

51 See, for instance: Mark Harrison, *Medicine in an Age of Commerce and Empire: Britain and its Tropical Colonies, 1660–1830* (Oxford: Oxford University Press, 2010); Erica Charters, *Disease, War, and the Imperial State. The Welfare of the British Armed Forces during the Seven Years’ War* (Chicago: University of Chicago Press, 2014).

52 Gavrus and Lamb, *op. cit.* (note 42). In the introduction to their book, Gavrus and Lamb mention several sociological or anthropological publications in English on contemporary medical education, to which the following book can be added: Emmanuelle Godeau, *L’« esprit de corps ». Sexe et mort dans la formation des internes en médecine* (Paris: Éditions de la Maison des Sciences de l’Homme, 2007). Social science approaches to medical education are alive and well, as evidenced by two recent English-language publications: Iveris Martinez and Dennis W. Wiedman (eds), *Anthropology in Medical Education. Sustaining Engagement and Impact* (Cham: Springer, 2021); Vania Smith-Oka, *Becoming Gods: Medical Training in Mexican Hospitals* (New Brunswick: Rutgers University Press, 2021).

53 Hans Pols, C. Michele Thompson and John Harley Warner (eds), *Translating the Body: Medical Education in Southeast Asia* (Singapore: National University of Singapore Press, 2017).

54 Lincoln C. Chen, Michael R. Reich and Jennifer Ryan (eds), *Medical Education in East Asia: Past and Future* (Bloomington: Indiana University Press, 2017), 16.

55 A pioneering work along similar lines is Ronald L. Numbers (ed.), *Medicine in the New World. New Spain, New France, and New England* (Knoxville: The University of Tennessee Press, 1987).

56 Cristiana Bastos, ‘Doctors for the Empire: The Medical School of Goa and its Narratives’, *Identities: Global Studies in Culture and Power*, 8, 4 (2001), 521.

57 In this sense, this special issue can be read in conjunction with this other recent special issue on the significance of medical history in medical education: Jacon Steere-Williams, Justin Barr, Claire D. Clar, Raúl Necochea López, ‘Remaking the Case for the History in Medical Education’, *Journal of the History of Medicine and Allied Sciences*, 78, 1 (2023), 1–8 (1–130 for the special issue it introduces).

