# Medical schools in empires: connecting the dots – CORRIGENDUM

**DOI:** 10.1017/mdh.2024.37

**Published:** 2024-07

**Authors:** Hohee Cho, Martin Robert

**Keywords:** Medical education, Medical school, Colonial medicine, Empire, Doctor, Medical profession, corrigendum

The authors apologise for an error in the article, which appears on page 114 of Volume 68, Number 2. In the 12th line of the page, a name has been misspelt as Job Arrizabagala and in footnote 15, as Jon Arrizabagala. It should be: Jon Arrizabalaga.
